# A Novel Fabry-Pérot Optical Sensor for Guided Wave Signal Acquisition

**DOI:** 10.3390/s20061728

**Published:** 2020-03-19

**Authors:** Cheng Xu, Zahra Sharif Khodaei

**Affiliations:** Department of Aeronautics, Imperial College London, London SW7 2AZ, UK; z.sharif-khodaei@imperial.ac.uk

**Keywords:** fiber optic sensors, Fabry-Perot, hybrid PZT-FBG, guided waves, structural health monitoring

## Abstract

In this paper, a novel hybrid damage detection system is proposed, which utilizes piezoelectric actuators for guided wave excitation and a new fibre optic (FO) sensor based on Fabry-Perot (FP) and Fiber Bragg Grating (FBG). By replacing the FBG sensors with FBG-based FP sensors in the hybrid damage detection system, a higher strain resolution is achieved, which results in higher damage sensitivity and higher reliability in diagnosis. To develop the novel sensor, optimum parameters such as reflectivity, a wavelength spectrum, and a sensor length were chosen carefully through an analytical model of the sensor, which has been validated with experiments. The sensitivity of the new FBG-based FP sensors was compared to FBG sensors to emphasize the superiority of the new sensors in measuring micro-strains. Lastly, the new FBG-based FP sensor was utilized for recording guided waves in a hybrid setup and compared to the conventional FBG hybrid sensor network to demonstrate their improved performance for a structural health monitoring (SHM) application.

## 1. Introduction

The increasing environmental concerns such as energy conservation and reducing life-cycle costs of engineering structures has led to the increasing use of composite materials in aeronautics and wind energy industries. Composite materials have a high strength-weight ratio and fatigue resistance and, therefore, their usage can reduce the fuel consumption and extend the aircraft’s service life. The application of composites in wind turbines can reduce the weight of the blade and, therefore, lead to an increase of the wind energy conversion rate and service time. However, composites’ anisotropic properties render them vulnerable to impact damage. Even a low-energy impact can cause various internal damage such as matrix cracking or delamination. These types of damage can lead to a catastrophic consequence if not detected.

Non-destructive inspection (NDI) methods such as radiography, ultrasound, magnetic testing, or visual inspections are involved and are widely used in the maintenance of engineering structures to ensure the integrity of composites. However, these technologies cannot perform a real-time monitoring of the structure in-service and require long down-times [1]. In wind turbine maintenance, the visual inspection takes place every 500 h while a detailed internal inspection with radio testing takes place annually. An internal crack can extend to a severe state between the annual maintenance check, which will lead to a decrease of the service time due to the damage. Given all the challenges of inspecting the state of any structure using NDI methods, structural health monitoring (SHM) has gained increasing attention in the inspection of engineering structures. A real-time SHM system can be regarded as a solution to solve the previously mentioned challenges. An SHM system monitors the structure’s status to ensure its integrity during operations and detect any damage before it becomes catastrophic. The concept of SHM comprise of having permanently distributed sensors on the structures to monitor its response during the operation and make a diagnosis about the state of the structure. There are numerous sensor technologies and methodologies [2,3,4], which have been developed and tested significantly in controlled laboratory environments and/or in simple structures. Some of the most commonly used transducers in SHM are piezoelectric transducers (PZT) [5] and fiber optic (FO) sensors [6,7]. Each comes with its own advantages and disadvantages. PZT transducers are excellent as exciters of guided waves. However, to monitor large areas, a network of transducers is required, which, on a large scale, can become unattractive due to the additional weight, wiring, connectors, and interference that they may bring to complex aircraft structures. There have been some solutions proposed to overcome these challenges by developing an integrated film with inkjet-printed wires [8]. On the other hand, FO sensors can be multiplexed in large numbers on a single fiber and, due to their advantages such as flexibility, immunity from electromagnetic interference, low weight, and small size, they can be largely integrated into the structure to form a network [9,10,11]. FO sensors are excellent passive sensors that can detect small changes in strain (hence, detect damage) as well as variations in environmental factors such as temperature by recording changes in the reflected light spectrum (intensity and wavelength). There are different types of FO sensors applicable such as fiber Bragg grating (FBG) and Fabry–Pérot Interferometer (FPI) sensors, which are each suited to a specific application. However, the response of FO sensors is also affected by the load in which the structure is experienced. When the external loading distribution is known and uniform (e.g., static structures), the effect of damage on the strain distribution can be reliably identified. However, when the external loading remains unknown and non-uniform, it is hard to distinguish the changes due to loading and due to damage only in the FO sensors’ response. Another challenge of the FO sensors is the sensor network density. The effect of damage on the sensor response is very local and the coverage area for each FBG is in terms of 10–20 mm [9]. This leads to a high requirement in sensor networks’ density. Some researchers have tried to overcome this problem by developing distributed FO sensors (Rayleigh Back-Scatter (RBS) sensors,) to form a high density sensor network [9]. However, issues with the resolution and the sensitivity of the distributed sensors remain, which requires further investigation.

One solution that addresses the challenge of unknown load distribution for damage detection is to use the FO sensors in an active sensing system where the structure is loaded with a known waveform, e.g., generated by a PZT actuator where the recorded response has an expected waveform as well. The frequency of the excited waves is much higher than the external loading. Therefore, it is easily distinguished by filtering the low-frequency signals out. By combining the FO sensors with PZT actuators, the advantages of both technologies are enhanced, and the number of PZT transducers significantly decreased, which leads to a reduction in the additional weight, wiring, connectors, and electromagnetic interference.

In 2004, Ogisu et al. [12] developed the first hybrid damage detection system, which combined PZT and FBG. However, due to FBG’s low sensitivity in Lamb wave signal acquisition in the proposed hybrid system, most of the FBG sensors failed to record any signal 10 mm away from the PZT actuator despite the actuation signal amplitude of ± 75V. Since then, many researchers have focused on the optimization of the hybrid system design and the FO sensors to improve their performance. Combining these two different sensor technologies comes with its own challenges. To detect damage reliably with any SHM system, the influence of damage on the signals must be much higher than environmental and operational effects on the signals. For guided waves travelling through complex structures, the effect of temperature and vibration cannot be ignored. Therefore, the signal to noise ratio (SNR) must be high. Moreover, the FO sensors are axial sensors, which means they are only sensitive to axial strain. Therefore, the sensitivity of the FO sensors to strain must also be high in order to capture the guided waves travelling in all directions in a structure.

Some researchers have focused on maximizing the wave transfer to the optical fiber by enlarging the strain transfer rate (STR) of the optical fiber. Sakai et al. (2016) demonstrated the enhancement of the FBG output response by using an acoustic lens [13]. The primary disadvantage to these approaches is that they add a relatively bulky device to the system and are, therefore, difficult to implement on a large number of densely-spaced FBG sensors. In addition, K. Peters has enhanced Lamb wave detection using FBG by converting Lamb waves into acoustic modes in an optical fiber through remote bonding [13]. However, there will be limitations for the application of such sensors to structures in-service.

This paper aims to develop a new hybrid PZT-FO solution that has higher sensitivity to strain signals and, therefore, is more reliable for damage detection in composite structures. To achieve this hybrid system, a novel FBG-based Fabry–Pérot (FP) sensor has been developed, which has higher strain sensitivity and, hence, higher SNR. This paper first starts with the principle of the hybrid system architecture, which is followed by the developments of the novel sensor by designing the sensor and validating its spectrum experimentally against the simulated results. Afterwards, the novel sensor is tested with the hybrid setup to detect guided waves and compare its strain sensitivity to the conventional FBG-based hybrid response. Lastly, the application of the new hybrid system to detect artificial damage on a composite plate is demonstrated experimentally.

## 2. Hybrid PZT-FO System Principles

**Figure 1:**
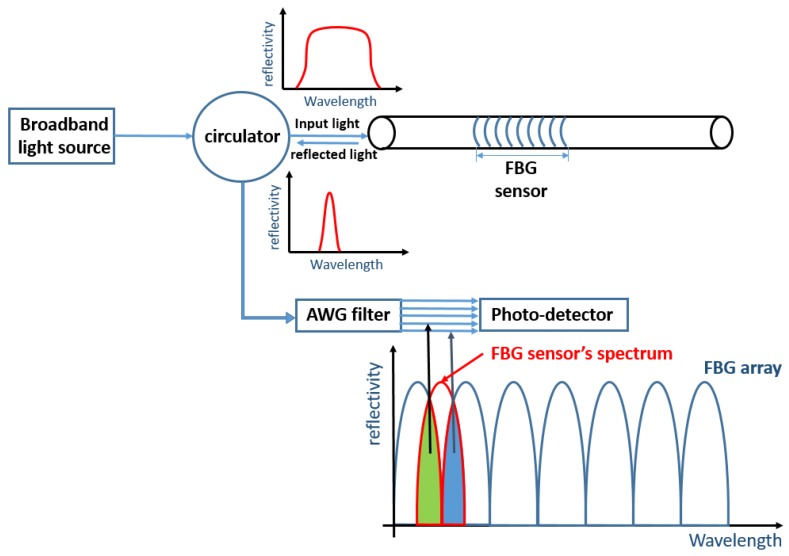
Principle of a power detection demodulation method [14].

Currently, there are two system architectures used in hybrid FBG measurement each with its own demodulation algorithm: power detection and edge filter detection demodulation [14]. In the power detection system, a broadband light source is used to deliver light to an array of FBG sensors. An arrayed waveguide grating (AWG) is used as a filter to demodulate the Bragg wavelength shift in FBG sensors by calculating the optic intensity fluctuations. The principle of this system is shown in Figure 1. [14]. This method has a multiplexing capability. However, since the broadband white light source has to distribute the optic intensity for each wavelength, its sensitivity is relatively low. Furthermore, a broadband white laser requires more power than a Tunable Laser Source (TLS), and long-time exposure under high optical power wears down parts of the system, which reduces the service life of the entire SHM system. In the power detection method, increasing the signal amplitude means increasing the shift of the overlap area between FO sensors’ spectrum and the AWG filter.

**Figure 2:**
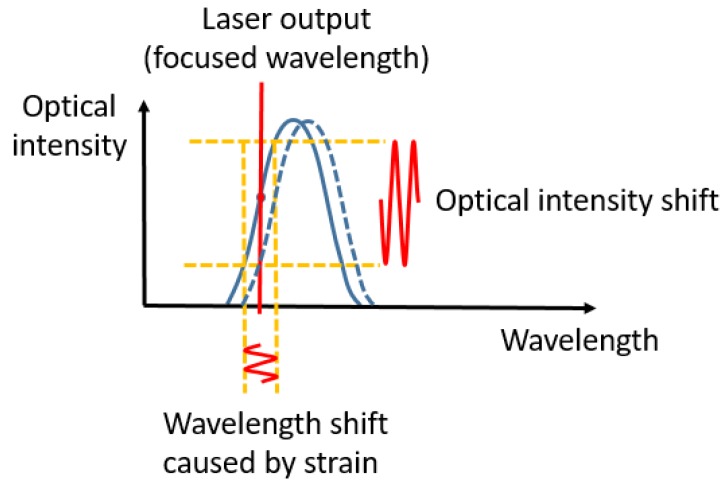
Principle of edge filter demodulation method [13].

In the edge filter detection method, a TLS is used. The wavelength of the TLS is adjusted to the middle of the FBG peak where the maximum slope is located. When the strain signal shifts the FBG sensors’ wavelength (due to propagation of an ultrasonic wave), the reflected light intensity changes as well, as depicted in Figure 2. [13]. These oscillations are recorded with a photo detector as a voltage signal. Compared to power detection, since all the input optic intensity is concentrated on a single wavelength, it has a higher SNR. However, because the laser and FBG sensors have a one-to-one correspondence, only one sensor can be demodulated at each time.

The hybrid system developed in this paper is based on the edge filter detection method to have a higher SNR.

## 3. Sensor Development

As described in the introduction, there are two main types of FO sensors.

**Figure 3:**
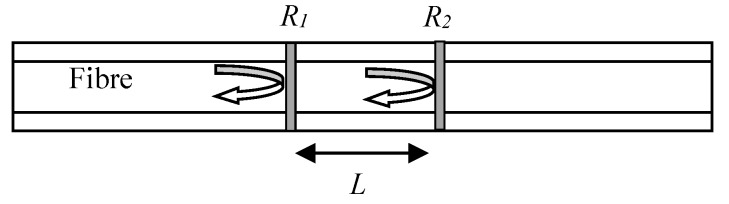
Principle of interferometric sensors.

**Figure 4:**
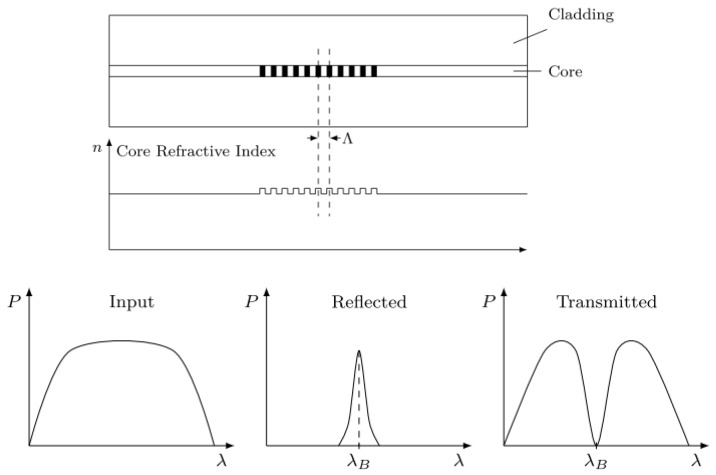
Principle of wavelength-based Fiber Bragg Grating (FBG) sensors.

- **Interferometric sensors** - There are four types of interferometric sensors [15]. Among them, the Fabry–Pérot Interferometer (FPI) sensor is the most popular one since, unlike the other types, it does not require a reference fiber with decreased efficiency. An FPI is generally composed of a pair of parallel partially reflective surfaces (with reflectivity *R_1_* and *R_2_*) separated by a certain distance (shown in Figure 3). Interference occurs due to multiple superpositions of the reflected beams from the two parallel surfaces. The light reflected from these two reflectors (mirrors) interfere with each other within the fiber. The strain and the temperature will change the length between the two mirrors, which will cause the phase shift between the two reflected lights. By monitoring the interfered light, the related strain and the temperature can then be detected. Through the interferometry, even a minute change in the sensing cavity length leads to a significant shift in the optical intensity. Compared to other types of FO sensors, interferometry-based sensors have higher sensitivity to strain and temperatures. However, most of them do not have the multiplexing capability or only have limited multiplexing. Some researchers have multiplexed sensors with a different cavity within one optic fiber and applied Fast Fourier Transform (FFT) to demodulate them since the sensors with different cavity will have a different frequency [16]. This method increased the number of sensors up to 8 per fiber but the length of each sensor varies, which can complicate the lamb wave signal demodulation.
- **Wavelength-based FO sensors**—As can be seen from Figure 4, when a beam of broadband light irradiates into the optical fiber, grating reflects a narrowband spectrum. The reflection occurs due to the unique structure of the FBG in which the refractive index of the core is periodically perturbed along the grating length. The reflection spectrum is centered at a characteristic wavelength called Bragg wavelength (λ), and is calculated based on the effective refractive index: $n_{eff}$ and the grating period Λ [17] as $\lambda =2n_{eff}\Lambda$. The strain and temperature change the distance between each grating, namely grating period, Λ. Therefore, by calculating the shifting of the Bragg wavelength (λ), the strain and temperature changes can then be demodulated. This demodulation method is called a wavelength-based demodulation method.

The objective of this work is to develop a new hybrid system for an SHM application with higher reliability in damage detection. To achieve this, the SNR of the FO sensors should be maximized: either by increasing the signal amplitude or decreasing the noise level [13]. In the wavelength-based demodulation method, to increase the FO sensor’s signal amplitude means to increase the wavelength shift caused by the same signal. However, in terms of the hybrid system, due to Lamb wave signal’s high frequency, it is impossible to use this method since there is not enough time to measure FO sensor’s whole spectrum. Both of the hybrid systems mentioned in Section 2 employ an optical-intensity-based demodulation method, which demodulate the strain by measuring the shift of optical intensity within a certain wavelength. In this case, to increase the FO sensors’ signal amplitude means to increase the reflectivity change caused by the same Lamb wave signal. For the purpose of the FO sensor development, the signal amplitude can be increased by optimizing the parameters of the FBG sensor, or by proposing a new or modified FO sensor.

### 3.1. FBG Optimization

One way to increase FO sensors’ signal amplitude is modifying the grating of an FBG to vary the ultrasonic wavelength-to-grating-length ($\lambda_{s}/L$) ratio [18,19,20]. Minardo [19] numerically predicted that the reflection modulation amplitude of an FBG at the edge slope significantly reduces with decreasing $\lambda_{s}/L$ from 6 to 1. The research reported in Reference [20] concluded that the ultrasonic wavelength, $\lambda_{s}$, must be at least six times the grating length, L, for the FBG response to be independent of the ultrasonic wavelength. Unfortunately, shortening the grating length also decreases the reflection intensity and, therefore, reduces the edge slope [21]. Furthermore, a non-uniform strain distribution within the FBG will lead to distortion in its spectrum, which makes it difficult to demodulate Lamb wave’s signal by focusing on one point in the spectrum [19].

### 3.2. Modified FO Sensors

For high frequency signals, a TLS is used as a light source, which increases the sensitivity to strain signals. According to the theory, however, the FBG length determines the upper limit for the detectable frequency and, to record high frequency signals, a short grating length is required. The short grating length results in a gentle slope at the edge of the FBG, which results in low sensitivity. To address this contradiction, Wu [22] proposed a phase-shifted FBG (PS-FBG) in a hybrid system to improve FO sensors’ sensitivity to the Lamb wave signal, which increased the SNR of the FO sensors. Compared to conventional FBG, PS-FBG sensors are designed to have a narrow valley in the middle of their spectrum. Wu [22] has demonstrated that the PS-FBG has higher sensitivity to detect high frequency small strain waves in comparison to FBG sensors. The length of the FBG has to be very small as a consequence of the higher sensitivity. Another drawback of the FBG sensors is their sensitivity to non-uniform strain distribution along the length of the grating [23] and distortion of the spectrum from a narrowband to a broadband, which will lead to problems in acquiring the guided wave signals through the edge reflection method.

Given the limitation of the previously mentioned techniques for enhancing the signal amplitude, a novel FO sensor has been developed in this study with the possibility of multiplexing. The proposed sensor is FBG-based FP to record guided wave signals from PZT actuators. Compared to FBG, the FBG-based FP sensor has a relatively higher sensitivity since the designed interference increases the signal amplitude and offers more freedom in the selection of sensing length since changing the sensing length will not affect the FO sensors’ spectrum and no distortion in the spectrum is expected when it is subjected to non-uniform strain distribution within the senor. The FBG-based FP sensors utilize the advantages of both type of sensors, but they need to be carefully designed. The development steps of this new sensor are detailed in Section 3.3.

### 3.3. Novel FBG-Based FP Sensor Development

For the SHM application, guided waves to be recorded are of high frequency, i.e., 50–300 kHz and the amplitudes are very small. This means that the optical intensity should be shifting, which is subjected to a very small strain. To achieve the higher sensitivity with the edge filter method, which is used in this research, higher slope in the spectrum is required (higher dI/dλ where I is the optical intensity and λ is the wavelength, which results in higher dI/dε). To develop an FBG-based FP (Fabry-Perot) sensor, optimum parameters such as cavity length, reflectivity, and Bragg wavelength of the sensors needs to be designed first. To achieve this, its spectrum has to be simulated accurately. To study the sensitivity of an FBG-based FP sensor in the lamb wave signal acquisition under the edge filter demodulation method, its spectrum should be obtained first and then one wavelength should be focused on to simulate the optical intensity shift caused by the change in strain due to the guided wave signals. Before simulating the FBG-based FP sensors spectrum, each FBG spectrum is simulated and then their interference is modelled given the FP cavity length.

Changing the cavity length in the FBG-based FP sensor does not cause any central wavelength shift, but will increase the interferometric fringe density within the wavelength bandwidth of its constituent FBGs. Although enhancing the interferometric fringe density will increase the optical power vs. wavelength shift slope, this relationship is not linear to each other, which is the case of the PS-FBG sensor [22]. This is something that can be optimized in our future research to obtain optimum sensitivity of the FBG-based FP sensor. 

#### Spectrum Simulation

An FBG sensor has series of parallel gratings printed into its core, which reflects a narrow wavelength of light when a broadband light is illuminated. Based on the reflection index distribution along the fiber axis, $n\left(z\right)=n_{0}+\Delta n_{max}\left[1+\gamma cos\left(\frac{2\pi }{\Lambda }z+\emptyset \left(z\right)\right)\right],$ the reflected spectrum of a uniform FBG sensor can be calculated by the equation below [24].

(1) $$R={\left|\frac{A_{2}\left(0\right)}{A_{1}\left(0\right)}\right|}^{2}=\frac{{\left|k_{12}\right|}^{2}sinh^{2}\left(\delta L\right)}{\delta^{2}cosh^{2}\left(\delta L\right)+{\left(\frac{\Delta \beta }{2}\right)}^{2}sinh^{2}\left(\delta L\right)}\;\frac{\Delta \beta }{2}=2\pi n_{eff}\;\left(\frac{1}{\lambda }-\frac{1}{\lambda_{B}}\right);\;\delta =\sqrt{k_{12}{}^{2}-{\left(\frac{\Delta \beta }{2}\right)}^{2}};\;k_{12}=\frac{\pi \gamma \Delta n_{max}}{\lambda }$$

where L represents the length of the FBG and γ represents the modulation depth of the Bragg grating. $\Delta n_{max}$ is the maximum change in the refractive index in the grating. $n_{eff}$ is the effective refractive index and Λ is the distance between each grating. $\lambda_{B}=2n_{eff}\Lambda$ is the center wavelength of an FBG.

The phase of the FBG is shown below.

(2) $$\phi \left(\lambda \right)=\{\begin{array}{c} \begin{array}{cc} \pi +\arctan\left[-\frac{\delta \cosh^{-1}\left(\delta L\right)}{\frac{\Delta \beta }{2}\sinh\left(\delta L\right)}\right] & \lambda \leq \lambda_{D} \end{array}\\ \;\begin{array}{cc} \arctan\left[-\frac{\delta \cosh^{-1}\left(\delta L\right)}{\frac{\Delta \beta }{2}\sinh\left(\delta L\right)}\right] & \;\;\;\;\;\lambda >\lambda_{D} \end{array} \end{array}$$

The fundamental equations leading to the above spectrum simulation can be found in Reference [24]. Before simulating the interference of two FBGs to form an FP sensor, the validity of the spectrum simulation is assessed by comparing the simulation results to an experimental spectrum. 

**Figure 5:**
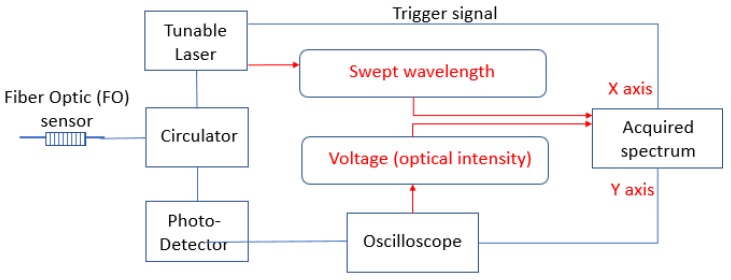
Experimental setup for a spectrum acquisition system.

The setup of the FO sensor’s spectrum acquisition system is shown in Figure 5. The tunable laser sweeps the wavelength range and irradiates a series of the light beam with different wavelengths in sequence. The irradiated wavelength array is regarded as the X-axis in the measured spectrum. The light beams go into the circulator and then transmits to FO sensors. FO sensors will reflect a certain optical intensity subjected to their corresponding wavelength. The reflected light travels through the circulator and then arrives at the photo detector. After the photo detector translates the optical intensity signal into the electric signal, the signal is then obtained by the oscilloscope. The acquired optical signal array is the Y-axis value of the spectrum. Combining the X-axis values (wavelength) and the Y-axis values (optical intensity), the reflected spectrum of the FO sensors can be acquired. 

**Figure 6:**
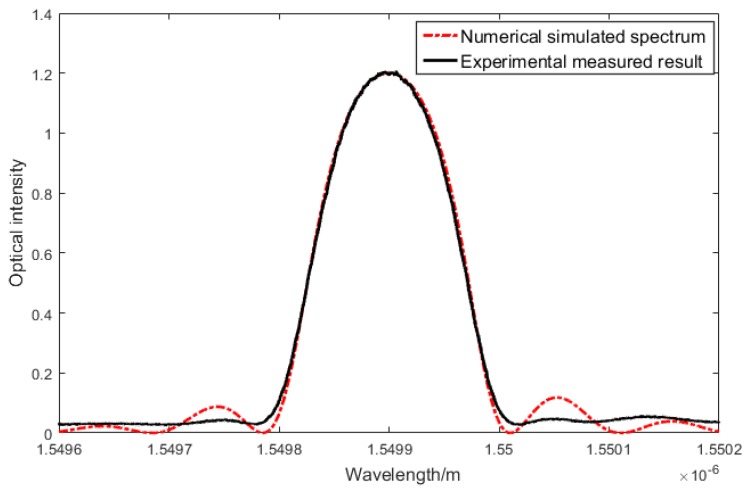
Fiber Bragg Grating (FBG) spectrum simulation, numerical vs. experimental results.

The details of the FBG sensor can be found in Table 1 and the results are shown in Figure 6, which shows a good agreement.

**Figure 7:**
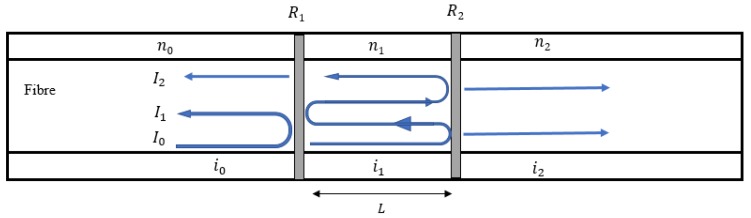
Light route in an FP (Fabry-Perot) cavity.

Once the spectrum for each FBG is obtained, FBG-based FP sensor’s spectrum can be simulated, according to the interference between the two spectrums as follows (shown in Figure 7).

Each reflected light ray can be calculated based on the reflectivity of each mirror and the cavity length. The whole reflected spectrum can then be simulated by adding all the reflected rays together. The final reflective spectrum for an FP cavity can be calculated by the equation below.

(3) $$R={\left|r\right|}^{2}=\frac{r_{01}^{2}+r_{12}^{2}-2r_{01}r_{12}\cos\delta }{1+r_{01}^{2}r_{12}^{2}-2r_{01}r_{12}\cos\delta }$$

where $r_{01}$ is the reflection coefficient when light travels from a space with a refractive index $n_{0}$ to a space with a refractive index $n_{1}$. $r_{12}$ is the reflection coefficient when light travels from a space with refractive index $n_{1}$ to a space with refractive index $n_{2}$. δ is the phase difference.

**Figure 8:**
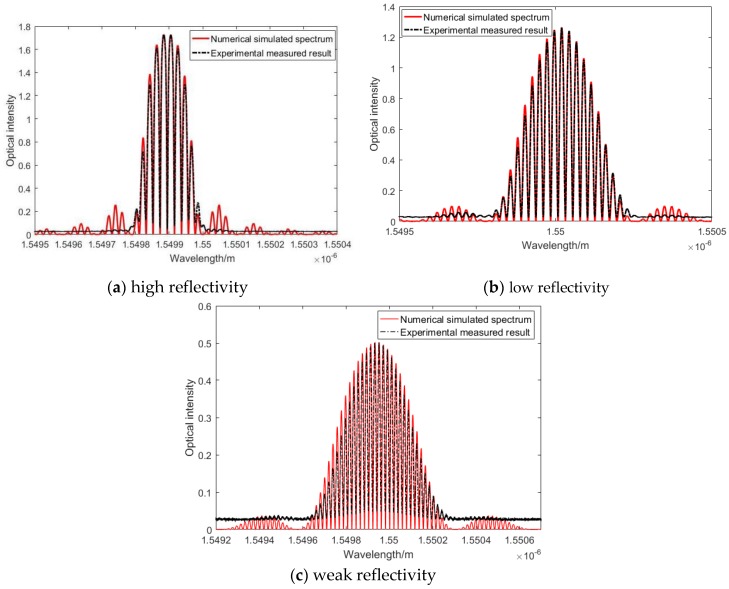
Comparison between simulated and measured FBG-based FP sensor’s spectrum.

Lastly, the FBG spectrum and the FP spectrum are combined to fine the interference pattern of the new FBG-based FP sensors.

(4) $$R_{F-P}={\left|r_{F-P}\right|}^{2}=\frac{{\left|r_{1}\right|}^{2}+{\left|r_{2}\right|}^{2}-2\left|r_{1}\right|\left|r_{2}\right|\cos\left(\beta h-\phi_{1}-\phi_{2}\right)}{1+{\left|r_{1}\right|}^{2}{\left|r_{2}\right|}^{2}-2\left|r_{1}\right|\left|r_{2}\right|\cos\left(\beta h-\phi_{1}-\phi_{2}\right)}$$

where $r_{1}\;\mathrm{and}\;r_{2}$ represent the reflectivity of the two FBGs that compose the FP cavity, β is propagation constant of light, and $\phi_{1}\;\mathrm{and}\;\phi_{2}$ are the phase of FBG1 and FBG2, which can be calculated from Equation (2). Figure 8a–c show the comparison between simulated FBG-based FP sensors and the measured FBG-based FP sensors with a different parameter in particular varying reflectivity. Note the change in optical intensity amplitude with different reflectivity parameters. Their parameters are shown in Table 2.

**Figure 9:**
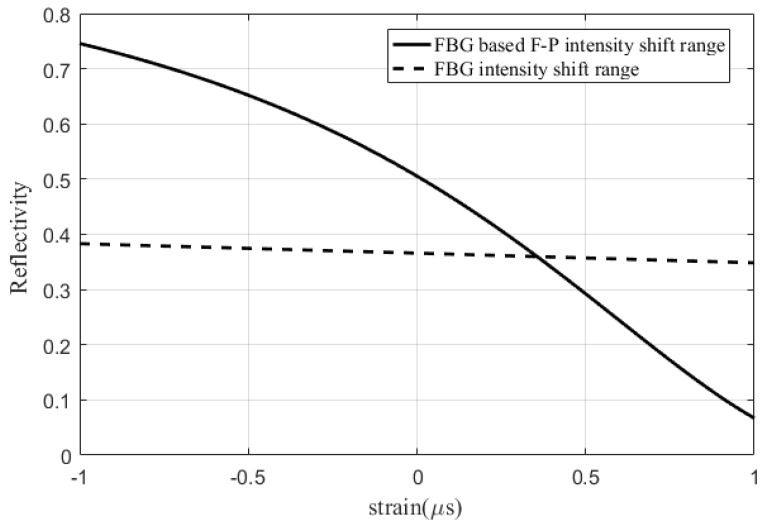
Sensitivity comparison between FBG-based FP sensor and FBG subjected to a micro strain.

Once the spectrum of the FBG-based FP sensor is obtained, the sensitivity of the sensor to a micro-strain under the edge filter method can be measured. This enables the demodulation between achieved optical intensity signal and the measured strain. The comparison between the FBG-based FP sensor and FBG’s signal amplitude under the edge filter method is shown in Figure 9.

Now that the FBG-based FP sensor spectrum can be simulated, the next step is manufacturing of the sensors, and designing an experimental set-up to test their applicability for a hybrid SHM application to record guided waves in a composite plate.

## 4. Experimental Procedure

To validate the application of the FBG-based FP sensor in detecting guided waves, an experiment was carried out where the guided wave response recorded by FBG-based FP was compared to the FBG hybrid set up. Afterward, the new hybrid system was tested in detecting artificial damage.

### 4.1. Sensor Manufacture

In an FBG-based FP sensor, the two FBGs acting as mirrors are required to have the same wavelength. The challenge, however, was due to manufacturing tolerances of wavelengths, which, for the annealing process, is around 0.15 nm but the required tolerance is 0.01 nm. If the challenge was not addressed, the two FBG’s spectrums will not have the required interference as simulated. To overcome this problem, a large number of FBGs with the same wavelength have been manufactured via a phase mask method separately by AtGrating Technologies. Then two of them with the closest wavelength were selected and spliced together to form an FBG-based FP sensor. The splice process led to a limitation of minimum cavity length. This is an issue that can be addressed with a different manufacturing process of the FBG.

### 4.2. System Architecture 

The working principle of optical strain measurements with an FBG-based FP sensor was based on reflectivity amplitude change. Hence, there were three steps to acquire Lamb waves: 1) Acquiring the reflectivity spectrum, 2) choosing a focus wavelength, and 3) measuring the change in the optical intensity at that focus wavelength.

**Figure 10:**
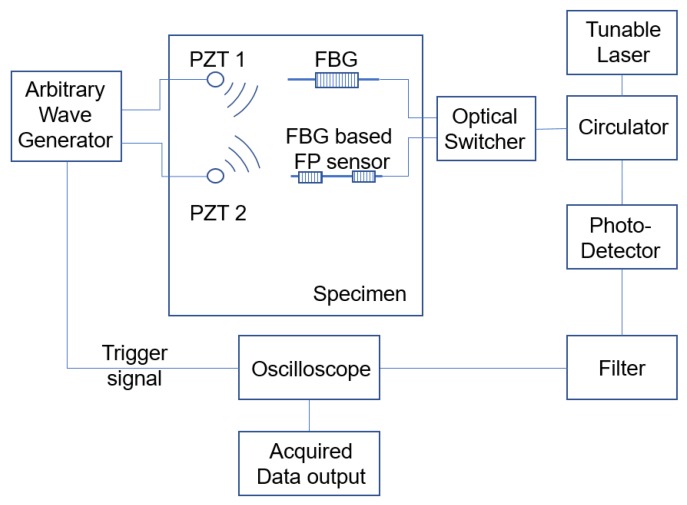
Schematic diagram of the hybrid system.

The hybrid system architecture is shown in Figure 10. First, the spectrum of each FO sensor at its current state was acquired in order to choose the suitable focus wavelength. The system setup for the spectrum acquisition was the same as the one mentioned in “Spectrum Simulation”. It consisted of a tunable laser, a circulator, FO sensors, a photo detector, and an oscilloscope. The tunable laser swept the wavelength range and irradiates light with a different wavelength to the circulator. The light beams are then be transmitted to FO sensors from the circulator. After FO sensors reflect the light with certain optical intensity, the reflected lights travelled through the circulator and reached the photo detector. The photo detector translated the optical intensity signal into electric signals, which is then recorded by the oscilloscope. Once the reflected spectrum is recorded for each sensor, the next step will be to choose the focus wavelength.

**Figure 11:**
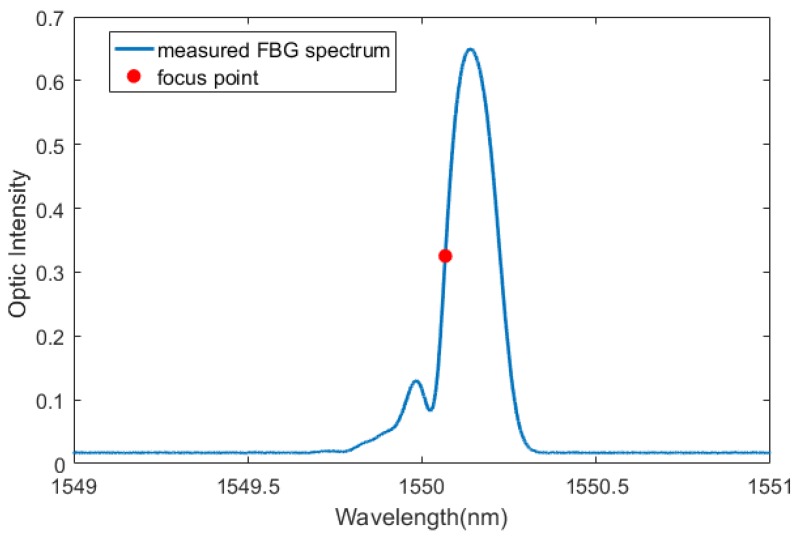
The FBG spectrum and the focus point for strain measurement.

**Figure 12:**
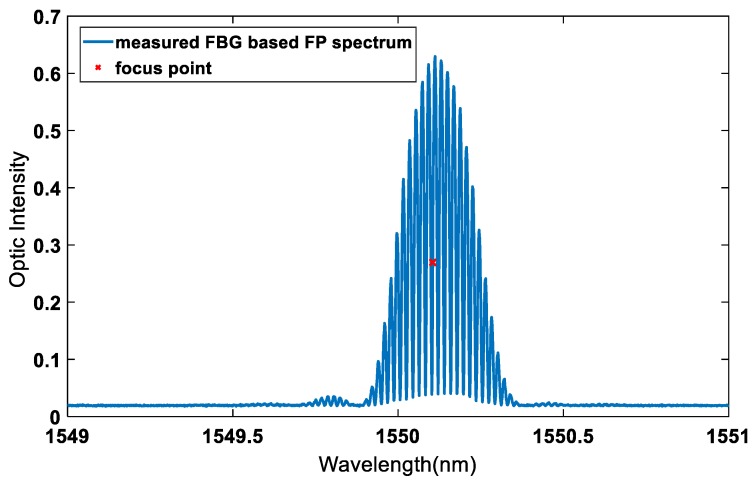
The FBG-based FP spectrum and the focus point for strain measurement.

To maximize the sensitivity of the sensor to small changes in strain, the focus point should be chosen at the largest slope of the optical intensity vs. wavelength curve (dI/dλ). The focus point was chosen as is half of the max optic intensity on the left side of the maximum slope (Shown in Figure 11 and Figure 12). After locating the focus wavelength, the Lamb wave signal was then acquired.

The arbitrary wave generator (AWG) was used to excite the PZT transducer. The photodetector was used to record the response of the FBG-based FP sensor. The light source was a tunable laser while the circulator isolated the light reflected from the FBG-based FP sensor to be measured by the photodetector. The intensity of the reflected light was spectrally filtered and converted to voltage. A trigger signal was sent from the arbitrary wave generator to the oscilloscope simultaneously after the excitation has started. The oscilloscope received the trigger signal and then started to record the data. On the other side of the system, the Lamb wave travelled through the structure and arrives at FO sensors. The fluctuation in the strain resulting from Lamb wave propagation, caused a shift in the reflected light intensity as well, which was then recorded by the photodetector. The acquired signal is then filtered and converted to voltage.

### 4.3. Specimen Preparation 

**Figure 13:**
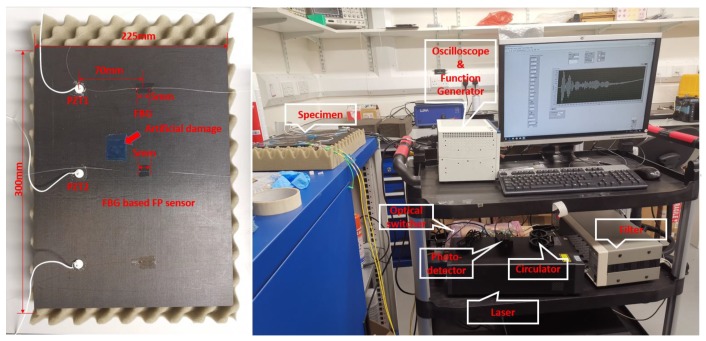
Experimental set up.

A carbon fiber reinforced polymer (CFRP) specimen was manufactured from prepregs (hexcel 914C-TS-5) as the host structure with the composite layup of: [0/45/−45/90/90/−45/45/0] _S_. The size of the specimen is 225×300×2 mm, the locations of the FBG sensor, the FBG-based FP sensor, the PZT actuator, and the artificial damage are shown in Figure 13.

### 4.4. Signal Acquisition—Pristine Structure

**Figure 14:**
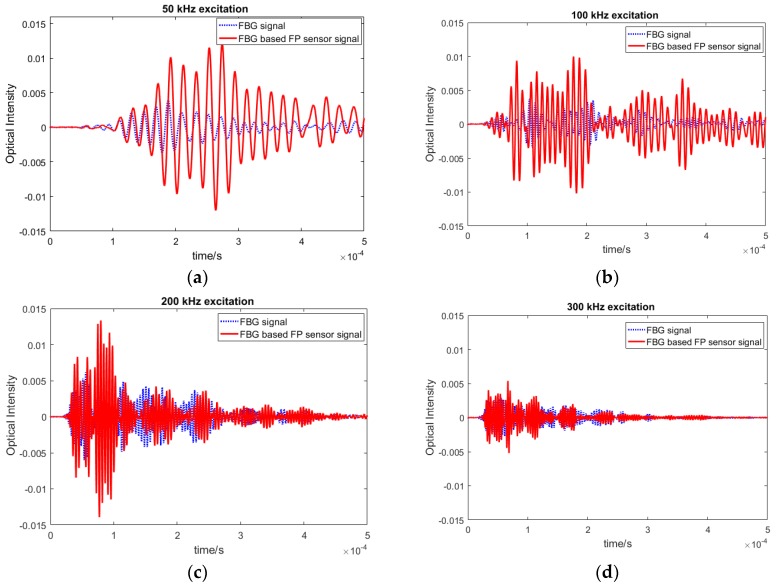
Comparison between FBG and FBG-based FP guided wave signals at (**a**) 50 kHz, (**b**) 100 kHz, (**c**) 200 kHz, and (**d**) 300 kHz excitation.

Once the focused point was determined, Lamb wave signals of different frequencies were actuated by the PZTs. In order to assess their strain sensitivity, i.e., amplitude of the recorded waves by the new FBG-based FP sensor, its response was compared with the similar signal recorded by FBG sensors, placed at the same distance as shown in Figure 13. There were two paths: PZT1-FBG-based FP sensor and PZT2-FBG sensor. The sensors were placed away from the boundary in order to isolate the effect of different boundary reflections. Both PZTs were actuated with the same waveforms and the same voltage and, therefore, they should share the same initial wave energy. The plate was excited by different frequencies from 50 to 300 kHz in order to compare different guided wave modes. The first anti-symmetric $A_{0}$ and symmetric $S_{0}$ modes, which were dominant at low and high frequencies, respectively. Each wave mode had a different characteristic, which made them suitable for detecting different damage types (e.g., surface damage or through thickness). Therefore, it was important to compare the responses of both sensors in recording different frequency responses. The FO sensor signals from the pristine specimen under different actuating frequencies are shown in Figure 14.

The results in different frequency excitations showed that the amplitude of the FBG-based FP sensors in recording lower frequency waves, i.e., anti-symmetric modes was noticeably higher than the FBG recorded guided waves. This means that the SNR for anti-symmetric modes was higher, which results in higher sensitivity and reliability in damage detection. To analyse the reliability of the FBG-based FP sensors, their responses under varying environmental effects have been investigated in the next sub-section. This was followed by simulating artificial damage on the plate, by adding mass on the plate, and by examining the residual signal (damage effect).

### 4.5. Environemnatl Effects on Recorded Signals

**Figure 15:**
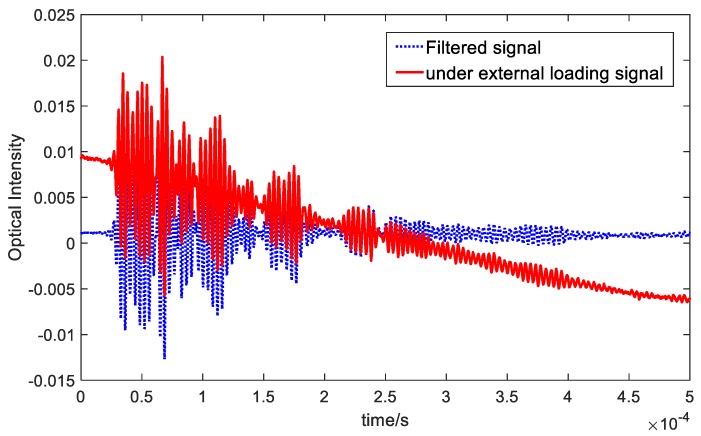
FBG-based FP sensor signals recorded under a varying external load on a pristine plate.

When the structure was monitored under varying environmental conditions, the noise (caused by the experimental set-up, vibrations, temperature effect, load, etc.) were different every time, which led to a difference in each measured waveform due to shifts in the optical intensity. According to the measurements, the environmental noise were within $2\times 10^{-4}$ amplitude. If the residual signal caused by damage was less than this order of magnitude, the damage cannot be detected reliably. However, when the residual signal shift was greater than the noise level, damage could be detected. To test the reliability of the FBG-based FP sensors to an environmental effect, guided wave signals under varying load were measured, as depicted in Figure 15.

**Figure 16:**
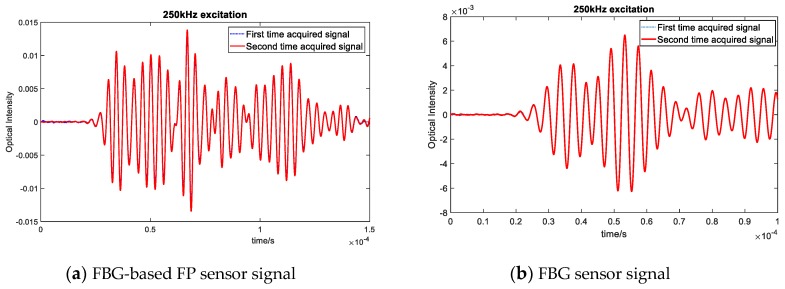
Processed data comparison between different environments.

The solid line within Figure 15 represents the Lamb wave signal recorded under an applied external load (in this example, an air flow was applied over the specimen). This caused the low frequency noise that can be seen in the recorded data. The method to remove environmental noise caused by this dynamic loading was to design and apply a band pass filter. The blue dashed line represents the filtered signal, which shows that the noise has been removed successfully. Subsequently, the sensor signals were recorded under different temperature and vibration load several times and filtered to see whether the effect of the varying operational conditions can be eliminated for both types of sensors. The filtered signals measured in different environments (under the same room temperature) are depicted in Figure 16 which shows that, for both FBG-based FP sensors and FBG sensors, the recorded signals after filtering matched very well.

### 4.6. Signal Acquisition—Simulated Damage

To study the sensitivity of the FBG-based FP sensors in detecting damage, their response under artificial damage were recorded and compared to the FBG sensor signals (which is the conventional hybrid set up). In this paper, for proof of concept, an artificial damage in the form of an additional mass was introduced onto the specimen. The added mass would change the propagation properties of the guided wave, which causes alterations to the signal in the form of refraction and reflection. Therefore, it could be used in the development stage as representative of damage in the composite plate. From the study on the pristine signals presented in Figure 14, it was observed that the response at different frequencies varies between the two sensors with the low frequency $A_{0}$ dominated wave modes (i.e., 50 and 100 kHz). These have higher amplitudes with the FBG-based FP sensor signals. Therefore, the effect of damage on these chosen frequencies are investigated in detail in this section.

**Figure 17:**
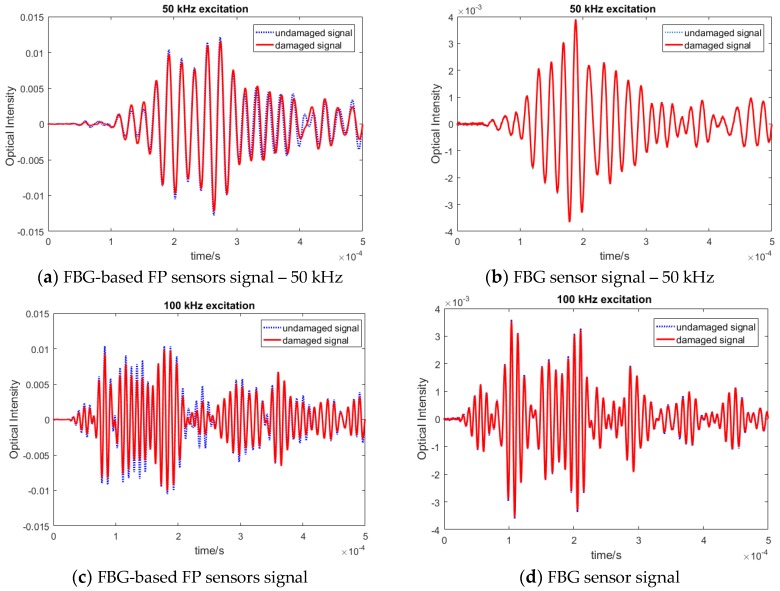
Comparison of damage signals recorded by (**a**), (**c**) FBG-based FP and (**b**), (**d**) FBG sensors.

**Figure 18:**
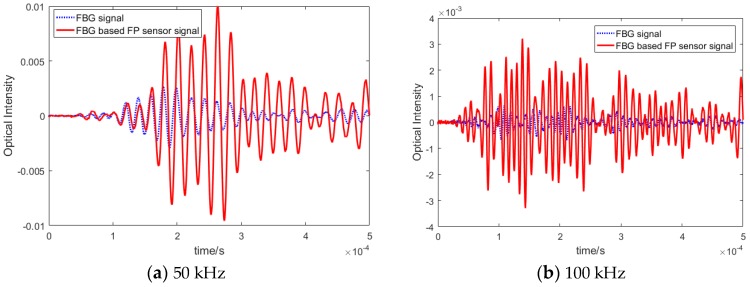

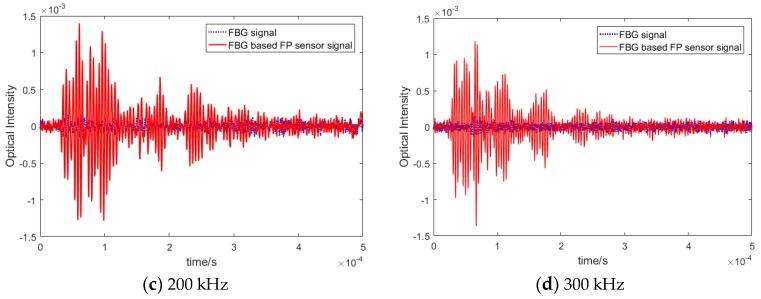
Comparison of a residual signal recorded by FBG-based FP and FBG sensors for different frequencies.

It can be seen that the effect of damage was much more pronounced on the FBG-based FP recorded sensor signals due to higher SNR and amplitude. Some of the selected frequency responses are shown in Figure 17. Similar behavior was also observed for higher frequencies. In particular, when the residual signal (i.e., pristine—damage state) was plotted, the higher amplitude of the FBG-based FP damage reflected signals was confirmed (see Figure 18). The results show that the FBG-based FP sensors could clearly record a small change in the guided wave propagation, which was due to damage (very small added mass in this experiment) in their direct path.

**Figure 19:**
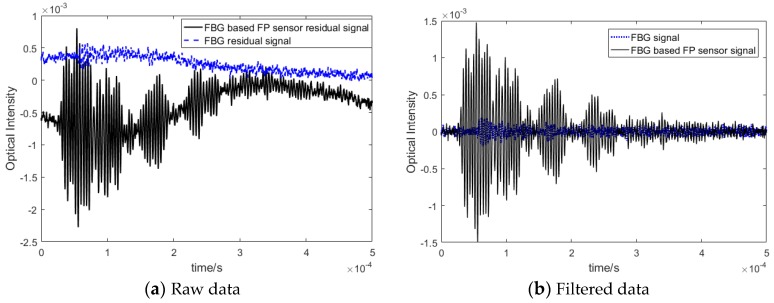
Comparison between FBG-based FP sensor and FBG residual signals under environmental conditions—250 kHz excitation.

In addition, to analyze the effect of a varying load on the reliability of the damage detection based on the recorded signals, the experiment was repeated by adding vibration (noise) as well as a random load to the structure. As the results in Figure 19 indicates, by applying a high pass filter to the signal, the effect of noise and external loading could be eliminated from the signals and the residual from FBG-based FP sensors remained above the noise level.

## 5. Discussion

In this section, the results of the FBG-based FP hybrid system are further analyzed to detect damage in composite plates. In the previous sections, it was shown that the residual signal from the proposed hybrid SHM system has high sensitivity to small changes in strain, and can be used to identify the presence of damage in the structure reliably. In this section, a damage detection algorithm based on the residual signal is proposed to quantify the reliability of the proposed methodology.

### Damage Index

To detect damage based on the guided wave signals three different damage indices are examined in this paper.

1. Normalized squared error between the signal and the baseline proposed by Michaels [25].

(5) $$DI_{1}=\frac{\int_{0}^{T}{\left[y\left(t\right)-x\left(t\right)\right]}^{2}dt}{\int_{0}^{T}x{\left(t\right)}^{2}dt}$$

where x(t) represents the pristine signal, y(t) represents the measured signal, and T represents the time window of the measurement.

2. The correlation coefficient between the signal and the baseline [26], which captures the changes in the shapes of the signals instead of the amplitude variations, which is the case for damage index 1.

(6) $$DI_{2}=1-\frac{\int_{0}^{T}\left[x\left(t\right)-\mu_{x}\right]\left[y\left(t\right)-\mu_{y}\right]dt}{\sigma_{x}\sigma_{y}}$$

3. The maximum amplitude of the residual envelope [5].

(7) $$DI_{3}=\frac{\max\left(\left|x\left(t\right)-y\left(t\right)-iH\left(x\left(t\right)-y\left(t\right)\right)\right|\right)}{\max\left(\left|x\left(t\right)\right|\right)}$$

where H(x(t)−y(t)) represents the Hilbert transform of the residual signal.

All three damage indices have been applied to the FBG-based FP recorded signals for the range of measured frequencies and compared to the conventional hybrid setup with the FBG sensor.

**Figure 20:**
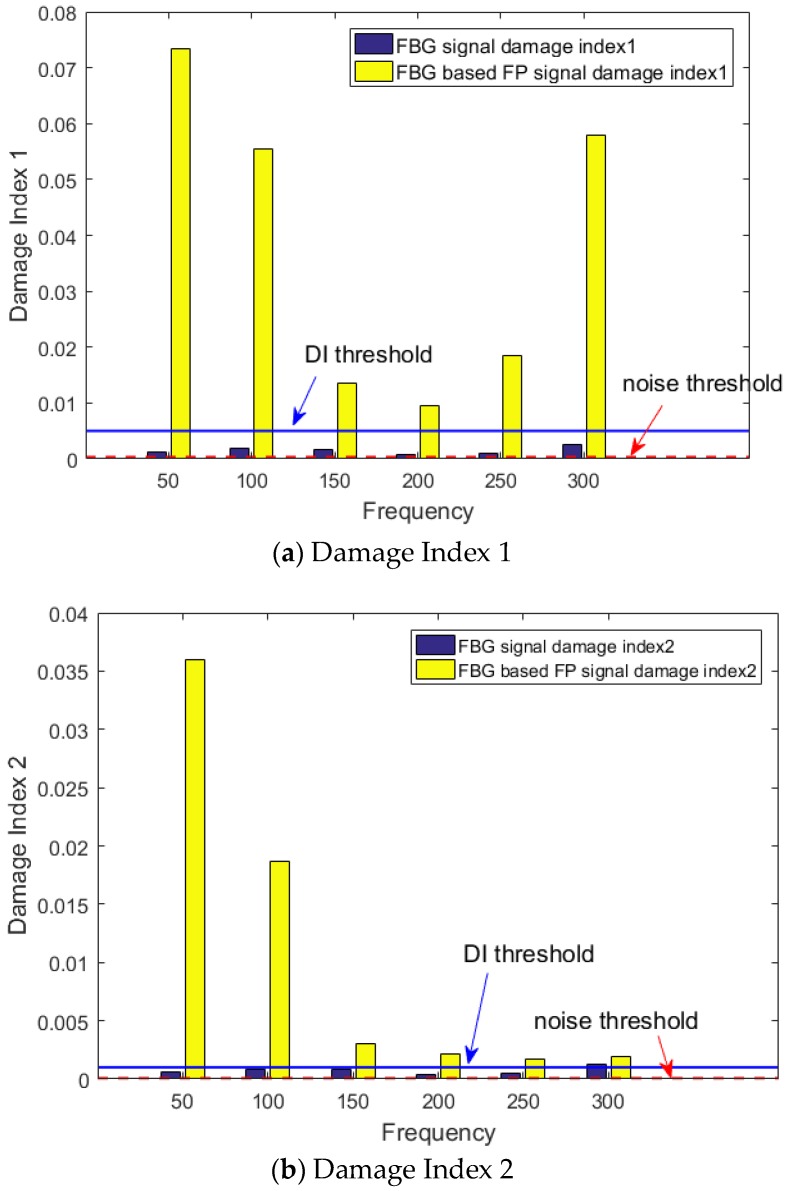

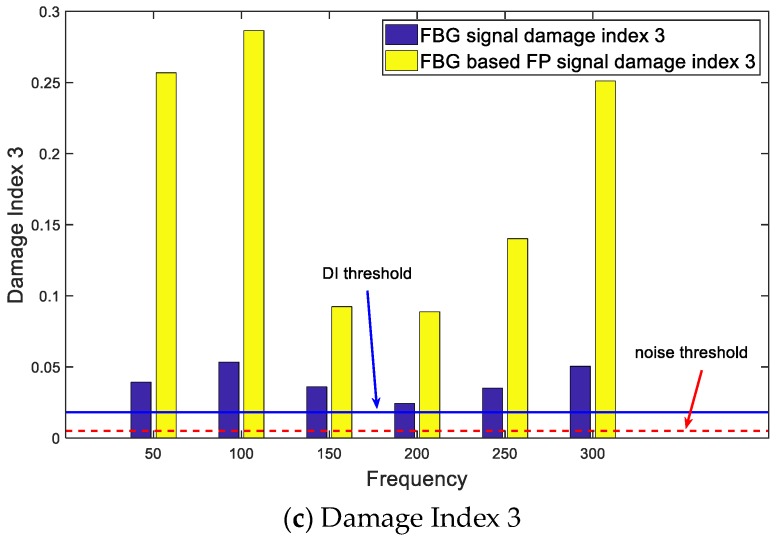
Different damage indices at various frequencies for damage detection.

From the results presented in Figure 20, it can be concluded that the anti-symmetric mode dominant at lower frequencies, such as 50 kHz, is more suited for detecting the artificial damage (added mass) in the composite plate. The DI values for all three methods are significantly above the threshold. This results in higher reliability for damage detection compared to the conventional hybrid solution, which is the FBG-PZT sensor system.

## 6. Conclusions

In this research, a novel FBG-based FP sensor was developed for an SHM system to monitor the integrity of composite structures and detect damage with high reliability under varying operational conditions. It was demonstrated that, by simulating the spectrum of the FBG-based FP sensors, a novel sensor with high sensitivity to small variations in strain can be designed and manufactured. The simulated FO spectrum was validated experimentally before the FBG-based FP sensor was integrated onto a composite panel for recording guided waves simulated by the PZT actuator. The FBG-based FP sensors demonstrated to have higher sensitivity to strain waves, i.e., higher intensity shift compared to traditional FBG sensors. In addition, under varying environmental conditions, such as varying noise level (vibration) and load in the structure, the pristine sensor signals recorded had high repeatability, which minimized the false alarm. This results in changes in the operational environment. When artificial damage was introduced in the structure, the FBG-based FP sensors residual showed to have higher amplitude was compared to the traditional FBG sensors for detecting guided waves. The lower frequency range such as 50 and 100 kHz were more reliable for detecting the added mass. This first experiment was a proof of concept, which showed the advantages of the novel FPG-FP sensor for SHM application in composite structures. The next step will be to test this new SHM system for detecting a barely visible impact damage (BVID) in a composite structure. Furthermore, the multiplexing capability of the sensor will be evaluated by developing more sensors on a single optic fiber with different central wavelengths. In addition, in the current set-up, only the FP cavity part in an FBG-based FP sensor is used for Lamb wave signal detection while the two FBG parts are not used for recording any response. The possibility of using them to measure the local temperature near the sensor to compensate the temperature effect on Lamb wave’s signal will be explored. Compared to FBG sensors, the spectrum shifting of each FBG-based FP sensor is within its own wavelength range. The multiplexing capability of the FBG-based FP sensors will also be evaluated in load monitoring and shape sensing.

## Figures and Tables

**Table 1:** Fiber Bragg Grating (FBG) sensor parameters.

Grating Length	Center Wavelength	Reflectivity	Refractive Index	Refractive Index Demodulation Depth
7.9 mm	1549.97 nm	71.75%	1.45	$1\times 10^{-4}$

**Table 2:** FBG-based FP sensor parameters.

Component /FBG Type	High Reflectivity	Low Reflectivity	Weak Reflectivity
Grating length	8.0 mm	3.5 mm	2.3 mm
Center wavelength	1549.894 nm	1550.017 nm	1549.941 nm
Reflectivity	50.17%	22.18%	18.93%
Cavity length	3.24 cm	3.18 cm	4.06 cm
Refractive index	1.45	1.45	1.45
Refractive index demodulation depth	$5.45\times 10^{-5}$	$1\times 10^{-4}$	$1\times 10^{-4}$
